# Metaverse technology tree: a holistic view

**DOI:** 10.3389/frai.2025.1545144

**Published:** 2025-04-17

**Authors:** Sepehr Ghazinoory, Fatemeh Parvin, Fatemeh Saghafi, Masoud Afshari-Mofrad, Nafiseh Ghazavi, Mehdi Fatemi

**Affiliations:** ^1^Department of Information Technology Management, Tarbiat Modares University, Tehran, Iran; ^2^Department of Systems Management and Decision Sciences, College of Management, University of Tehran, Tehran, Iran; ^3^Research School of Management, Australian National University, Canberra, ACT, Australia; ^4^Department of Industrial Engineering, Tarbiat Modares University, Tehran, Iran; ^5^Department of Technology and Innovation Management, University of Tehran, Tehran, Iran

**Keywords:** Metaverse, Metaverse technologies, technology tree, roadmap, meta-synthesis

## Abstract

**Introduction:**

The Metaverse has emerged as a significant trend in recent years, offering solutions across diverse fields. Despite substantial investments and extensive research efforts, a comprehensive understanding of the Metaverse environment and its full potential remains elusive. This article seeks to address this gap by developing a technology tree for the Metaverse based on published standards, prior studies, and frameworks proposed by leading firms.

**Methods:**

To construct the Metaverse technology tree, a systematic literature review approach was employed. From an initial pool of 354 scientific papers, conference proceedings, book chapters, and reports, a rigorous screening process –focused on titles, abstracts, and full-texts –resulted in a selection of 81 final sources. These sources were synthesized using a meta-analysis methodology.

**Results:**

The meta-synthesis of the selected literature produced a comprehensive Metaverse technology tree encompassing seven key branches: artificial intelligence, Mirror World, extended reality, network infrastructure, lifelogging, blockchain, and the Internet of Things. Each branch represents a critical technological area necessary for the development and realization of the Metaverse.

**Discussion:**

The proposed Metaverse technology tree offers a holistic overview and roadmap of the technological domains underlying the Metaverse. By identifying these seven branches, this research provides valuable guidance for future studies and development trajectories in Metaverse technologies.

## 1 Introduction

Technical knowledge, advanced technologies, and innovations in computer science are the most essential and fundamental capabilities required for participating in the global arena (Salamzadeh et al., 2022) as they transform and enrich human interactions, communications, and social transactions (Liu et al., 2023). Sean Parker—the founder of Napster and one of the co-founders of Facebook—claimed that the next human evolution after migrating from farms to cities would be toward the internet. With Mark Zuckerberg unveiling a preview of the Metaverse, the prophecy seems more achievable than ever as human life is on the brink of a historical transformation. Many large corporations are investing in the infrastructure of various virtual worlds (e.g., Decentraland, Sandbox, and CryptoVoxels), seeking to build exclusive hardware and software ecosystems to attract users and become their chosen destination in the Metaverse (Yemenici, 2022). McKinsey predicts that by 2030, most live events will be held in the Metaverse (especially education, development, and other collaborative activities), impacting 80% of the economy and generating over $5 trillion in added value. This massive impact is one of the main drivers of the development of Metaverse-related technologies (Coccia and Watts, 2020).

As the Metaverse employs various types of technology, analyzing it with technical, economic, marketing, and ecosystem perspectives requires identifying all related technologies to provide a holistic view of the concept. Although identifying all Metaverse technologies remains a significant challenge because of their evolving and dynamic nature, presenting a “big picture” of its technical dimensions and components would be useful. Therefore, a reference model will help identify, compare, and evaluate the Metaverse ecosystem and predict future technology trends (Lee et al., 2015). Scholars have presented various methodologies for technology identification.

Focusing on the activities that form firms’ value chains, the technology value chain methodology maps each activity to one or more technologies or technology areas (Porter, 2011). The process-based methodology defines firms’ processes as coordinated activities that convert inputs into outputs with added value or valuable results for customers (Hammer and Champy, 2009). As technologies are utilized in various activities (e.g., core, support, and management activities) (Mili et al., 2009), core technologies are identified according to the firm’s activities (Mansouri et al., 2021). Highlighting customer needs, the quality function deployment (QFD) methodology identifies technologies that deliver products/services according to the required qualities (Song et al., 2015). The technology mapping methodology focuses on the relationships between technologies in a specific technology field or industry, using various tools (e.g., text, tables, or diagrams with nodes and connecting arcs) to display these relationships. In a diagram, each node represents a topic, concept, application, or any other information, while the arcs illustrate their relationships. The output of technology mapping is usually a list of general technologies applicable to various products in diverse industries (Gudanowska, 2016).

**Figure 1:**
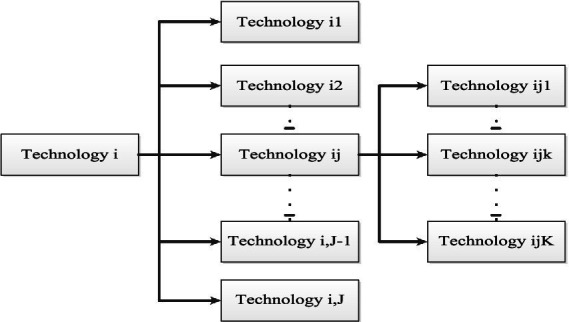
Structure of a technology tree.

The technology tree methodology provides an overall panorama of technologies in a specific field, which is very suitable for understanding these technologies and their relationships. A hierarchical approach is used in this method, with the number of levels depending on how detailed the technology is examined (Heiss and Jankowsky, 2001). The technology tree is a valuable tool for identifying existing and emerging technologies in a field, representing technological features (e.g., functions, goals, and components) in a hierarchical structure. The central idea behind the technology tree is to identify, classify, and organize the critical components of technologies in a field. Therefore, the technology tree is vital in developing technology strategies built on specialized knowledge and requires significant resources (Qiu and Wang, 2023; Tseng, 2008). However, specialized knowledge may not be broad enough in some fields to reflect the rapidly changing and diverse technology features. Moreover, as the structure of the technology tree may vary depending on the experts, an objective viewpoint is required to develop the technology tree (Chang et al., 2009). The technology tree is a chart that effectively presents information about technological features (Choi et al., 2012). Although it can be developed for various purposes and take different forms, the most common structure is hierarchical, including information about technologies and their relationships (or technological structures) in a target field. Thus, defining the technologies and analyzing their hierarchical relationships is the key to developing a technology tree. Figure 1 illustrates the structure of a technology tree, indicating that technology i (i = 1,…,I) consists of technology ij (j = 1,…,J) and technology ijk (k = 1,…,K). A technology tree can be considered a type of technology ontology. A recent trend in ontology is to automatically generate ontologies from large datasets such as web data (Morente-Molinera et al., 2019; Rani et al., 2017), biomedical data (Xiang et al., 2015), or building data (Lee et al., 2016).

Given the lack of a comprehensive study in previous Metaverse research, this article proposes a Metaverse technology tree based on related published models and standards, previous studies, proposed solutions from leading Metaverse enterprises, and the examination of other scientific documents. Other scholars can consider the proposed technology tree as a foundation and adapt it for their studies. Additionally, this technology tree can be used to identify the technical capabilities of Metaverse products and services, providing a valuable technical resource for policymakers. The rest of the article is structured as follows. Section 2 reviews the literature and related works, Section 3 describes the research methodology, Section 4 discusses the proposed technology tree, and Section 5 concludes the article.

## 2 Literature review

Although the Metaverse notion attracted attention in 1984 with William Gibson’s cyberpunk novel “Neuromancer,” the term “Metaverse” or “3D world” was first coined in 1992 in Neal Stephenson’s novel “Snow Crash,” which depicted a three-dimensional hyper-reality world consisting of avatars of real people. This concept expanded in the following decades through computer games and entered a new phase in the 2000s with the “Second Life” game, which offered an early form of virtual world interaction (Lynch, 2012; Lee et al., 2021). The Metaverse does not have a precise and unique definition and is evolving and changing. As technology advances and people encounter new experiences in the virtual world, the concept and definition of the Metaverse gradually expand. However, this article uses the definition proposed by the Metaverse Standards Forum: An inclusive and networked virtual environment for human and digital interaction, encompassing technologies, internet-enabled objects, and individuals interacting in a virtual world. This hybrid environment includes real and virtual worlds that individuals access through various devices, including sensors, virtual reality, augmented reality, and other artifacts (Smart et al., 2007).

The Metaverse utilizes capabilities from a wide range of technologies, and their categorization requires effort. Various studies have examined this field from different perspectives and proposed components and enabling technologies for the Metaverse (Table 1). However, a comprehensive reference is needed for a more precise categorization of Metaverse technologies. Although few studies have been conducted on the Metaverse, and those that have provided categorizations of Metaverse technologies focused only on a specific technology branch, no study has covered all the different branches of Metaverse technologies. To address the research gap, this article uses a top-down approach to propose a technology tree for the Metaverse that covers all related technology fields.

**Table 1:** Metaverse components and technologies.

References	Title	Enabling components and technologies
Smart et al. (2007)	A cross-industry public foresight project	Lifelogging, augmented reality, virtual worlds, Mirror Worlds
Sami et al. (2023)	The Metaverse: Survey, trends, novel pipeline ecosystem, and future directions.	(1) Hardware and equipment, (2) frameworks, libraries, and platforms, (3) avatar and object modeling, (4) environment rendering, (5) user sessions and authentication, (6) user-to-user interaction, (7) user interaction with the business, and (8) in terms of relevant topics and various aspects of the technologies related to the Metaverse (a) artificial intelligence, (b) blockchain, (c) networking, (d) computing, (e) business, (f) privacy and security, and (g) ethics.
Mozumder et al. (2022)	Overview: Technology roadmap of the future trend of Metaverse based on IoT, blockchain, AI technique, and medical domain Metaverse activity	Blockchain, artificial intelligence, Internet of Things, and digital twins
Wang et al. (2023)	A survey on the Metaverse: The state-of-the-art technologies, applications, and challenges	Network infrastructure: The network and communications infrastructure required for the Metaverse Technology management: Technologies related to the management and control of the Metaverse environment Common core technologies: Core technologies, including cloud computing and artificial intelligence Connecting virtual reality objects: Technologies related to objects and tools that are connected to the Metaverse Virtual reality convergence: Technologies required for the fusion of real and virtual worlds in the Metaverse
Sun et al. (2022)	Big data meets Metaverse: A survey	Big data, artificial intelligence, virtual reality, augmented reality, mixed reality, and other technologies will diminish the difference between online and real-life interaction.
Tsou and Mejia (2023)	Beyond mapping: Extend the role of cartographers to user interface designers in the Metaverse using virtual reality, augmented reality, and mixed reality	Extended reality, including virtual reality, augmented reality, and mixed reality
Yun and Leem (2021)	An industry-service classification development of the Metaverse platform	virtual reality, augmented reality, and digital twin
Al-Ghaili et al. (2022)	A review of Metaverse’s definitions, architecture, applications, challenges, issues, solutions, and future trends	Blockchain, virtual reality, augmented reality, artificial intelligence, 3D reconstruction, Internet of Things, edge computing
Lee et al. (2021)	All one needs to know about Metaverse: A complete survey on technological singularity, virtual ecosystem, and research agenda	Extended reality, user interactivity (human-computer interaction), artificial intelligence, blockchain, computer vision, the Internet of Things and robotics, edge and cloud computing, and future mobile networks
Kayid (2020)	The role of artificial intelligence in future technology	Neural networks, genetic algorithms, symbolic artificial intelligence, deep learning, quantum machine learning, hierarchical reinforcement learning, Bayesian deep learning, affective computing, automated machine learning, generative adversarial networks

## 3 Methodology

This research employs a systematic review approach. This methodology was chosen because it allows us to identify the most relevant and high-quality studies on the topic and provide a quantitative analysis of the research trends and patterns (Tavana and Sorooshian, 2024). Initially, research keywords (including the Metaverse, technologies, map, survey, and roadmap) were developed to identify studies aligned with the research objective. According to the systematic review process guidelines, the keywords were searched in the title, abstract, and keywords of articles indexed in Web of Science and Scopus, using the “advanced search” to ensure inclusion criteria (Table 2).

**Table 2:** Search strings in databases.

Database	Search string
Web of Science	TS = (Metaverse AND technologies AND (map OR survey OR roadmap))
Scopus	TITLE-ABS-KEY (Metaverse AND technologies AND (map OR survey OR roadmap)) AND (LIMIT-TO (DOCTYPE, “ar”) OR LIMIT-TO (DOCTYPE, “cp”) OR LIMIT-TO (DOCTYPE, “ch”) OR LIMIT-TO (DOCTYPE, “le”))

The 354 sources included journal articles, conference papers, book chapters, and reports (Table 3). Given the importance of the quality and credibility of the sources, only 226 journal articles were reviewed further, of which 33 and 23 were excluded because of duplication and content irrelevance, respectively. After evaluating the content of the chosen 170 articles to exclude those not focused on Metaverse technologies, the final 81 articles were studied thoroughly.

**Table 3:** Distribution of identified sources using the systematic review method.

Source type	Number of sources
Journal articles	226
Conference articles	104
Book chapters	12
Reports	1
Total	354

## 4 Findings

**Figure 2:**
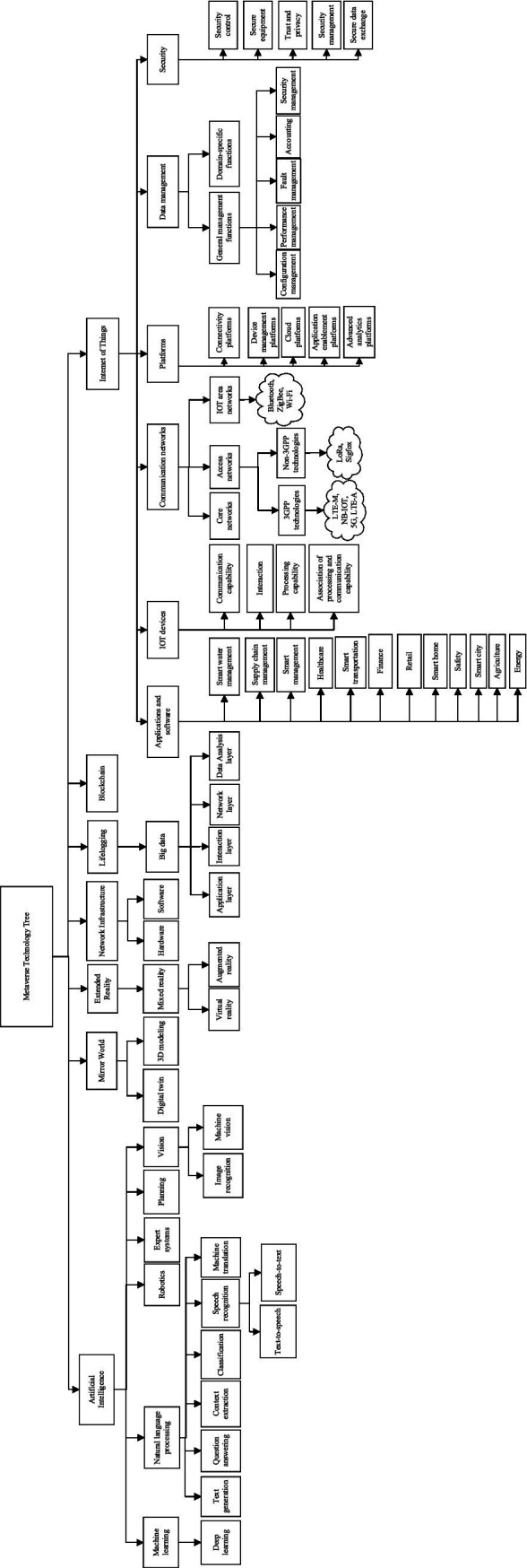
The Metaverse technology tree.

Figure 2 illustrates the proposed Metaverse technology tree, constructed using the findings from the final articles. The tree includes seven main branches of technology (Figure 1)—artificial intelligence, Mirror World, extended reality, network infrastructure, lifelogging, blockchain, and the Internet of Things—each of which is discussed below.

### 4.1 Artificial intelligence (AI)

Artificial Intelligence is a transformative technology that plays a crucial role in developing the Metaverse. By enhancing user experiences, AI facilitates deeper and more realistic interactions within virtual environments. It enables computers to engage intelligently with users, allowing seamless communication and interaction. Furthermore, AI can learn from experiences and data, empowering it to create new and unique content tailored to individual preferences. Additionally, AI can understand and utilize different languages, making virtual experiences more accessible to a diverse audience. Consequently, AI creates an intelligent, interactive, and advanced virtual environment that continuously adapts to users’ needs and preferences (Kayid, 2020; Fadhel et al., 2024).

#### 4.1.1 Machine learning

Machine learning is a branch of AI where computers learn from data, identify patterns and relations, predict according to the information, and use previous and current data for performance improvement and intelligent decision-making. This process allows computer systems to constantly self-optimize and react better to new circumstances by analyzing input data. In particular, deep learning is a specific machine learning method used to achieve further advancements in data processing and pattern recognition, especially in image and natural language processing (Xu et al., 2021).

#### 4.1.2 Natural language processing (NLP)

NLP allows machines to understand and interact with human language by handling various language-related tasks, from answering questions and classifying texts to conversing with users (Khurana et al., 2023).

- Text generation is a powerful tool for mimicking human language patterns and styles according to the data-driven and AI-powered world (Celikyilmaz et al., 2020).
- Question answering focuses on responding to questions structured in natural languages.
- Content extraction converts unstructured text into structured data for more straightforward analysis, allowing machines to understand and automatically process human language (Arasti and Moghaddam, 2010).
- Classification builds models to predict new data labels based on previously labeled data, managing and enhancing online conversations (Yun and Leem, 2021).
- Machine translation interprets between different languages, using models to improve conversations on social platforms, facilitate text comprehension, and help cross-lingual communication within various organizations. The best machine translation systems can identify words with multiple meanings and determine the language of each word (Stahlberg, 2020).
- Speech recognition enables computer systems’ perception and interpretation of human speech. Through various NLP algorithms and models (Gaikwad et al., 2010), this process facilitates more natural and effective interactions between users and the virtual environment. Speech recognition is classified into text-to-speech and speech-to-text:
- Text-to-speech (TTS) converts written text into artificial speech by analyzing it to understand its meaning and then generating a vocal version (Gaikwad et al., 2010). In the Metaverse, TTS facilitates reading messages and announcements to present information to users and enhance accessibility to various audiences.
- Speech-to-text (STT) converts audio signals into text by recognizing sounds, analyzing speech patterns, and generating coherent text (Gaikwad et al., 2010).

#### 4.1.3 Robotics

As a critical field within AI and computer science, robotics focuses on designing, building, and programming robots or automated devices with capabilities similar to human movement and functionality in the virtual Metaverse world (Grischke et al., 2020).

#### 4.1.4 Expert systems

Expert systems are computer programs designed to model the decision-making ability of a human expert in a specific field. These programs mimic human decision-making by identifying logical patterns experts use (Turban, 1992). Expert systems facilitate user–system interactions, optimize resources, predict and prevent issues, and manage the virtual economy, enhancing the overall user experience in the Metaverse.

#### 4.1.5 Planning

Planning in AI involves designing sequences of actions that allow systems to achieve specific goals in real or virtual environments (Turban, 1992).

#### 4.1.6 Computer vision

Computer vision combines visual and textual data to enhance understanding of texts and their meaning (LaValle, 2006). Computer vision has two sub-fields:

- Machine vision processes two-dimensional images, enabling computers to observe and analyze the surrounding environment. Machine vision equips a computer with several advanced cameras and a powerful analytical system to process visual data.
- Image processing enables computers to see and interpret images and video content better. Autonomous vehicles, traffic violation cameras, and facial recognition systems rely on image processing to function accurately.

### 4.2 Mirror world

Mirror World is an innovative technology that creates digital representations of real environments, objects, and systems. This technology plays a significant role in the Metaverse by enabling users to interact with realistic simulations of the physical world. Mirror World has extensive applications across various domains, including education and training, infrastructure management and maintenance, product design and development, commerce and marketing, urban management and planning, and environmental and energy management. By using the Mirror World, organizations can enhance educational processes, reduce operational costs, optimize design workflows, improve customer experiences, and manage physical resources more effectively (Szeliski, 2022).

#### 4.2.1 Digital twin

Digital twin constructs precise digital models of physical objects and systems as a starting point for digitizing physical environments. Reflecting periodic changes in their virtual counterparts, digital twins generate digital replicas of physical environments, creating “multiple virtual worlds” (Kye et al., 2021).

#### 4.2.2 3D modeling

3D modeling helps develop digital twins in Mirror World to simulate and analyze various realities, providing more accurate information about physical objects and environments (Ranathunga et al., 2023).

### 4.3 Extended reality (XR)

Extended reality (XR) combines various technologies to provide a hybrid experience of virtual reality (VR), augmented reality (AR), and mixed reality (MR) in the Metaverse (Sacchi et al., 2020). XR utilizes all these elements to enable users to interact within virtual environments, experience more realistic scenarios, and engage with physical objects and information (Tsou and Mejia, 2023). In the Metaverse, XR creates simulated environments, virtual workspaces, entertainment, and digital games.

#### 4.3.1 Mixed reality (MR)

MR is a subset of XR that blends VR and AR to bridge the gap between the virtual and augmented worlds. In the Metaverse, MR integrates the digital and real worlds into a single environment (Braud et al., 2022; Yun and Leem, 2021).

- Augmented reality (AR) adds virtual elements to the real world; users see the physical world through their devices’ cameras, overlaying it with virtual information (e.g., text, images, or 3D objects) (Hoang et al., 2023).
- Virtual reality (VR) transports users to a completely virtual world where they cannot notice the surrounding physical world (Turner, 2023; Hoang et al., 2023).

### 4.4 Network infrastructure

The network infrastructure is a foundational technology that connects and coordinates devices and computers, enabling the transmission of data at high speeds while minimizing latency. In the context of the Metaverse, this infrastructure is essential for managing the significant volume of data required for extended reality (XR) experiences. It must support real-time interactions that involve heavy graphics between users, ensuring a seamless and immersive experience. The network infrastructure is also critical in maintaining data security and ensuring stable connections, which are vital for fostering user trust and engagement in the Metaverse (Nasrin et al., 2016).

#### 4.4.1 Hardware

Network infrastructure hardware—including routers, switches, cables, and servers—facilitates data transmission, creating and supporting a stable, high-performance network by ensuring fast and effective communication between devices (Turban, 1992).

#### 4.4.2 Software

Network infrastructure software incorporates operating systems, protocols, and security software that manage and control network resources (Nasrin et al., 2016). These software solutions ensure real-time communications, data security, and network stability while also optimizing and protecting the network against cyber threats.

### 4.5 Lifelogging

In the Metaverse, “lifelogging” refers to the digital recording and preservation of an individual’s life. Utilizing advanced technologies and tools, lifelogging continuously and automatically captures information about daily activities, experiences, locations, social interactions, and physiological states, generating vast amounts of big data (Smart et al., 2007). This searchable digital history allows users to review and interpret their experiences, leading to a deeper understanding of individual behavior and reactions. Furthermore, lifelogging facilitates the development of digital memories and enhances overall lifestyle by providing insights that can enhance decision-making and personal growth.

#### 4.5.1 Big data

A subset of lifelogging, big data refers to collecting, storing, and analyzing large and complex data generated by individuals to record details of their lives and personal experiences. Big data has four layers acting as its sub-technologies:

- The interaction layer facilitates engagement between users and the Metaverse, using virtual information and real-world data to allow users to see and interact with virtual objects in the physical environment in the MR context (Sun et al., 2022).
- The application layer creates and executes various applications in the Metaverse, handling tasks ranging from entertainment and education to commercial services (Qazi et al., 2022).
- The network layer establishes and manages communications between devices and services in the Metaverse (including virtual reality devices), ensuring accurate and secure data transfer (Sun et al., 2022; Liu et al., 2018).
- The data analysis layer analyzes big data collected from the Metaverse and other sources through preprocessing, data mining, model design, model evaluation, and results presentation. This layer transforms data into usable and meaningful information for various purposes (e.g., recommendations, product preprocessing, pricing, and security management) (Qazi et al., 2022; Liu et al., 2018).

### 4.6 Blockchain

Blockchain technology provides a reliable framework for recording and transferring information and transactions between digital objects and individuals. In the Metaverse, blockchain enables decentralized verification of ownership, secure value transfer, enhanced transparency, and seamless connectivity (Liu et al., 2018). The encryption offered by blockchain allows users to maintain control over their data and simplifies the transfer of AI-related ownership agreements. Additionally, blockchain enhances privacy through zero-knowledge proofs, a cryptographic method that allows users to verify information without revealing sensitive data, enabling them to manage their data securely. Its ledger provides a transparent audit trail for verifying transaction accountability. Furthermore, blockchain ensures digital identity management, enhancing the security and validity of data and transactions while also helping to protect against deepfakes by ensuring the accuracy of information.

### 4.7 Internet of things (IoT)

The Internet of Things (IoT) is a pivotal technology in the Metaverse, enabling a wide array of objects—such as sensors, AI devices, and network-connected electronics—to connect and share information. This connectivity facilitates automated interactions between objects and people, as well as between objects and their surrounding environments (Mansouri et al., 2021). Within the Metaverse, IoT allows for remote monitoring, control, and management of these interconnected devices. The data collected through IoT interactions serve as critical inputs for performance optimization and informed decision-making, ultimately enriching the user experience and operational efficiency in virtual environments (Huynh-The et al., 2023).

#### 4.7.1 Applications and software

The Internet of Things includes applications and software for managing and analyzing data in various fields within the Metaverse’s sub-branches. These sub-branches include smart water management, supply chain management, healthcare, smart transportation, assets, finance and investment, retail, smart home and cities, safety, agriculture, and energy, all of which contribute to advancing these sectors.

- Smart water management: Utilizing sensors and IoT technologies for optimized water resource monitoring and management, helping create sustainable virtual environments.
- Supply chain management: Enhancing efficiency and transparency in the supply chain through inventory monitoring, shipment tracking, and process optimization.
- Smart healthcare: Connected devices and sensors monitor users’ conditions and provide remote healthcare services, facilitating health-oriented interactions.
- Smart transportation: Optimizing transportation systems in the Metaverse through virtual traffic monitoring, fleet management, and real-time information provision (ITU, 2012).
- Assets, finance, and investment: Improving the Metaverse’s asset management, investment, and financial processes through more precise monitoring and data-driven decision-making.
- Retail: Enhancing customer engagement, optimizing inventory management, and streamlining sales processes to create a seamless virtual shopping experience.
- Smart home: Automating and controlling home devices in the Metaverse (e.g., lighting, heating, security, and entertainment) via the Internet (Jamshidi et al., 2023).
- Safety: Monitoring and controlling safety devices and systems (e.g., CCTV cameras and warning sensors) (Xue et al., 2023).
- Smart city: Enhancing efficiency and quality of life in virtual Metaverse cities through traffic monitoring, waste management, street lighting, and other urban services (Zallio and Clarkson, 2022).
- Agriculture: Using sensors and connected devices to monitor and manage agricultural processes (Kusuma and Supangkat, 2022).
- Energy: Monitoring and controlling energy devices and systems to optimize consumption and reduce costs (Rao and Sandhu, 2024).

#### 4.7.2 IoT equipment

IoT connects the physical and virtual worlds to provide advanced services using efficient information and communication technologies (Grischke et al., 2020). Therefore, the equipment branch covers all physical IoT objects or devices with sensing and activation capabilities. These devices are classified into four categories based on communication and processing capabilities (Gaikwad et al., 2010; LaValle, 2006):

- Communication capability determines how a device can connect to a network branch. Connectivity in IoT equipment addresses connecting and exchanging data between different devices through wireless or wired networks. These devices can use Wi-Fi, Bluetooth, Zigbee, LoRa, and NB-IoT protocols to continuously collect, process, and transmit data to central servers or other devices, which helps develop an intelligent and connected network.
- Interaction describes how devices communicate with each other. For example, data collection and transport devices (e.g., infrared readers and barcode scanners) read or write data from physical objects. Sensor/activator devices—like thermometers—interact directly with physical environments, collecting data from them. These devices interact, process data, and send information to other devices for decision-making and operation, enhancing efficiency and accuracy.
- Processing capability determines whether the devices can perform part of the processing locally (at or near the device itself) and collect data. This feature helps reduce latency and the volume of data transmitted to servers while improving energy efficiency. In traditional IoT models, the cloud was responsible for most of the processing, but with the increase in the number of devices, edge computing has become essential to perform calculations close to the data source.
- The association of processing and communication capabilities highlights that although a combination of high/low processing capabilities and high/low communication capabilities is possible, low-processing power devices rarely have sufficient communication capabilities, as some processing power is required for communication.

#### 4.7.3 Communication networks

Communication networks include two pivotal capabilities: network capabilities to control connection performance (e.g., resource control, access control, and accounting) and transport capabilities to provide IoT data connectivity (Grischke et al., 2020). Three types of networks—core, access, and IoT area—play vital roles in the network branch (Zhang and Liu, 2023):

- The core network connects the provider domain with the access network, enabling long-distance communications.
- The access network connects devices in the physical IoT branch to the core network, divided into 3GPP and non-3GPP:
- 3GPP technologies, particularly 4G and 5G, can transmit data at high speeds but are costly in terms of hardware, maintenance, and energy consumption. Therefore, various standards (e.g., LTE-A, LTE-M, and Narrowband-IoT (NB-IoT)) are introduced to address these disadvantages. LPWAN wireless technologies cover wide-range communications with low transmission speeds and small packet sizes, prolonging battery life.
- Non-3GPP technologies refer to networks that do not follow the third-generation partnership project (3GPP) communication standards for cellular networks (e.g., 4G and 5G), with Wi-Fi, LoRa, Sigfox, and Bluetooth as notable examples. These networks are widely used in IoT alongside 3GPP networks, especially for low-bandwidth, long-range, or low-power communications.
- IoT area networks connect devices within a limited area through Bluetooth, ZigBee, and Wi-Fi. Facilitating communication between devices nearby, these networks are suitable for smart homes and buildings (Zhang and Liu, 2023).

#### 4.7.4 Platform

A platform consists of technological components that deliver specific functions and services to support applications (Stahlberg, 2020). IoT platforms are divided into connectivity, device management, cloud, application enablement, and advanced analytics platforms (Khurana et al., 2023):

- Connectivity platforms manage the network components of IoT systems, ensuring stable device connections.
- Device management platforms ensure each device is connected and secure, update device statuses and software, report changes in device status and measured data, and manage security updates.
- Cloud platforms provide the infrastructure required for coordinated IoT systems and offer scalable IoT solutions.
- Application enablement platforms offer integrated solutions that encompass devices, software, and deployment support for IoT systems.
- Advanced analytics platforms offer complex IoT analytical systems focusing on machine learning, AI, and statistical models for analyzing large datasets (Arasti and Moghaddam, 2010).

#### 4.7.5 Management capabilities

IoT data management involves efficiently handling the vast amounts of data IoT devices generate by collecting, storing, processing, and analyzing data to extract insights and support decision-making. The key aspects include general management functions and domain-specific functions (Akpakwu et al., 2017):

- General management functions include monitoring the overall strategy, planning, and coordinating data-oriented activities by defining proper data governance frameworks, ensuring data quality and integrity, managing data lifecycles, and enforcing data security and compliance policies (Ge and Li, 2010). The International Telecommunication Union (ITU) categorizes general management capabilities into five functional classes: fault, configuration, accounting, performance, and security management (Mansouri et al., 2021).
- Fault management identifies, diagnoses, and resolves issues or failures in IoT systems and networks by monitoring device performance, detecting anomalies or errors in data transmission or device operation, and initiating appropriate responses (e.g., alerts, notifications, and automatic corrective actions). By addressing issues impacting data integrity, system availability, or overall performance, fault management ensures IoT systems’ continuous operation and reliability in the Metaverse (Diène et al., 2020).
- Configuration management involves systematically managing and controlling IoT devices and systems’ settings, parameters, and configurations. By managing device configurations, firmware updates, and network settings, this function ensures stability, accuracy, and optimal performance, facilitates scalability and flexibility in IoT deployments, and enables centralized management of diverse devices and configurations while adhering to security and operational standards (Li et al., 2020).
- Accounting focuses on the financial aspects of data management in IoT by tracking and allocating costs associated with IoT deployments (e.g., hardware, software, and operational expenses). As a critical subset of general management, accounting also ensures compliance with financial regulations and standards while providing insights into the profitability and cost-effectiveness of IoT initiatives in the Metaverse (Samaniego and Deters, 2017).
- Performance management optimizes IoT system efficiency by tracking key performance indicators (KPIs), such as data throughput, latency, reliability, and system uptime (Chen et al., 2019).
- Security management protects IoT devices, networks, and data from unauthorized access, breaches, and cyber threats by implementing robust authentication, encryption, and access control measures that safeguard sensitive information and ensure data integrity (Eyada et al., 2020).
- Domain-specific functions focus on data aggregation and contextualization, which are crucial for filtering and organizing large amounts of sensor data collected from IoT devices. These functions aim to facilitate efficient data storage, retrieval, and utilization for decision-making processes in specific Metaverse applications (e.g., smart cities, healthcare, and industrial automation).

#### 4.7.6 Security capabilities

As IoT evolves, addressing security risks becomes increasingly critical (Negash et al., 2017). Given the role of each discussed sub-branch in collecting, transmitting, and analyzing IoT data, these security issues can affect all of them, posing severe challenges. Therefore, a set of security capabilities is associated with all branches, with the most critical capabilities divided into five categories (Zhang et al., 2014):

- Security control refers to specific measures and mechanisms implemented to protect devices, networks, and data from various threats through technologies, policies, and procedures designed to enforce security requirements and mitigate risks. Examples include encryption for data transmission, authentication mechanisms for device access, secure software updates, and intrusion detection systems. Robust security controls are essential to protect the Metaverse environment from unauthorized access, data breaches, and other cyber threats, ensuring IoT deployments’ overall integrity and reliability (Ford et al., 2023).
- Secure equipment refers to hardware devices incorporating built-in security features to protect against vulnerabilities and threats, including IoT sensors, actuators, gateways, and other connected devices designed with robust security mechanisms (e.g., hardware-based encryption, secure boot processes, and tamper-resistant components) (Ali et al., 2019).
- Trust and privacy ensure that users and stakeholders trust their data security and responsibility management by establishing transparent policies and practices for data collection, storage, and sharing and implementing privacy-preserving techniques (e.g., anonymization, consent management, and data encryption to protect individuals’ private information) (Sicari et al., 2015).
- Security management involves comprehensive planning, implementation, and monitoring of security measures through risk assessments, threat modeling, and security policy development to protect IoT devices, networks, and data from cyber threats. It also incorporates continuous monitoring, incident response planning, and regular updates to mitigate vulnerabilities and ensure system resilience against evolving security challenges (Chen, 2017).
- Secure data exchange ensures that data transmitted between devices, networks, and systems is protected from interception, tampering, and unauthorized access by implementing encryption protocols (e.g., TLS/SSL) for secure communication channels, ensuring data integrity through message authentication codes (MACs), and using secure APIs and protocols (e.g., MQTT with security enhancements) for reliable information exchange. Security measures prevent malicious actors from exploiting vulnerabilities in communication channels and protect sensitive information throughout the Metaverse (Sukiasyan et al., 2022).

## 5 Discussion

The Metaverse, as an emerging virtual-physical experience, integrates diverse technologies to create interactive virtual environments with applications ranging from entertainment to specialized fields (Herath et al., 2024). Beyond offering new user possibilities, the Metaverse also introduces new capabilities for various industries. However, the Metaverse remains in its early stages, with major technology giants (e.g., Microsoft, Apple, Google, Facebook, and gaming studios) seeking to harness its potential for growth and application.

Despite the Metaverse’s diverse technological landscape (Table 1), existing studies have not comprehensively addressed all its aspects. For example, Wang et al. (2023) examined Metaverse technologies, providing an analysis of their current state, related policies, and technical and practical aspects. Therefore, this article offers a comprehensive and systematic analysis of the Metaverse, presenting a detailed roadmap encompassing its technologies and applications. This approach aligns with the results of Mozumder et al. (2022), who examined the future technological roadmap of the Metaverse based on IoT, blockchain, AI, and the Metaverse activities in healthcare, emphasizing the challenges and ambitious plans of tech giants for the realization of the Metaverse.

This article introduces a comprehensive Metaverse technology tree (Figure 2), organizing technologies into multiple hierarchical levels (Figure 1). Compared to closely related research, the seven dimensions offer a more complete analysis of the Metaverse’s technology and applications (Table 4). The proposed Metaverse technology tree can encompass other technologies and expand in specific areas by adding more details to each technology branch. As the Metaverse evolves, it will transform human interactions with virtual environments and influence multiple technological fields.

**Table 4:** Comparison of the article with previous studies.

Authors	Title	Key results
Wang et al. (2023)	A survey on the Metaverse: The state-of-the-art, technologies, applications, and challenges	Examining the current state of the Metaverse and related policies Presenting the Metaverse roadmap Reviewing the Metaverse’s technical and practical aspects
Mozumder et al. (2022)	Technology roadmap of the future trend of Metaverse based on IoT, blockchain, AI technique, and medical domain Metaverse activity	Investigating the future technologies of the Metaverse Assessing the environmental impacts of the Metaverse Presenting the Metaverse applications in the medical field Reviewing the Metaverse technologies
Cheng (2023)	Metaverse	Defining the pivotal concepts of the Metaverse Analyzing the Metaverse’s impact on social interactions and lifestyle Evaluating the Metaverse’s impact on distance education Reviewing privacy and inclusivity challenges
Massetti and Chiariello (2023)	The Metaverse in medicine	Investigating new and emerging technologies Reviewing medical applications of the Metaverse Analyzing the Metaverse with a futuristic approach
Al-Ghaili et al. (2022)	A review of Metaverse’s definitions, architecture, applications, challenges, issues, solutions, and future trends	Reviewing the Metaverse inclusivity Presenting a proposed framework for the Metaverse Analyzing future challenges and trends Representing the Metaverse technologies graphically
Lee et al. (2021)	All one needs to know about Metaverse: A complete survey on technological singularity, virtual ecosystem, and research agenda	Providing a comprehensive framework for the Metaverse Analyzing the Metaverse key technologies Proposing a research agenda for the Metaverse Examining social and moral issues in the Metaverse
This article		Defining critical concepts comprehensively and accurately Analyzing key and emerging technologies Reviewing practical and innovative applications of technologies in the Metaverse Illustrating the Metaverse technology tree Forecasting and presenting possible trends

## 6 Conclusion

The technology tree is a potent data structure in computer science that enables efficient searching, modification, and retrieval of information while providing hierarchical categorization for improved system performance. A well-structured Metaverse technology tree enhances its development and management. One of the essential advantages is increased transparency and a better understanding of the Metaverse; by having an overview of all existing technologies and systems, developers and managers can quickly identify the strengths and weaknesses of each section and anticipate and implement the best technology strategies and development roadmaps. This comprehensive view also aids in resource optimization, as a better understanding of needs and available capabilities allows for more efficient allocation and management of resources. The Metaverse technology tree is crucial for coordinating and communicating among various technologies, enabling better synchronization in complex and multifaceted projects. Moreover, by increasing flexibility in response to rapid changes and developments in the technology world, organizations can quickly adapt to innovations and market changes, leveraging new opportunities. Thus, the Metaverse technology tree is critical for adapting to future changes and advancements.

Despite offering a comprehensive Metaverse technology tree to aid decision-making, this study faced limitations, including data scarcity, challenges in transferring results across environments, precise technology selection, and defining the Metaverse accurately. Additionally, the correct interpretation of results and the validation of the Metaverse technology tree are the limitations that can be investigated in future research. Enhancing Metaverse applications may involve training computers to interpret physical movements for better virtual interaction. Furthermore, future research could explore training headsets and sensors to increase control. Moreover, solving Metaverse challenges requires a large amount of data. Since real-world data collection is time-consuming, synthetic data generation can improve speed and accuracy. Examining how to provide synthetic data for the Metaverse could also be an interesting research subject.

Moreover, given the evolution of its software and hardware space, the Metaverse concept needs to be clarified. Therefore, the Metaverse needs to be expanded in both software and hardware dimensions, with scholars examining the obstacles to the Metaverse development and possible solutions based on the conditions of each geographic location. Bias in interpreting Metaverse data highlights the need for guidelines ensuring ethical behavior among avatars and users. To guarantee the interests of all users, relevant experts should review these rules and ethical frameworks to eliminate or at least reduce the misuse of the Metaverse for the benefit of individuals and large firms. Regarding transferability, a massive investment is required to ensure proper interaction between the Metaverse and the physical world, between humans and the Metaverse, and between hardware and software. Beyond the steady expansion and regular updates of popular platforms (e.g., Second Life and Roblox), the avatar and the individual should be continuously connected, increasing the speed and authentication of transfers. Ensuring such a permanent connection could be a promising topic for future research.

## Data Availability

The original contributions presented in the study are included in the article/supplementary material, further inquiries can be directed to the corresponding author.
